# Lung Tissue Metabolome Investigation Reveals the In Vivo Effects of Zhuye Shigao Decoction in an LPS‐Induced Acute Pneumonia Model in Mice

**DOI:** 10.1155/mi/6034066

**Published:** 2026-05-11

**Authors:** Haipeng Tang, Zhiliang Sun, Weibo Qin, Guangzhi Cai, Wenyi Gao, Yang Wang, Jiyu Gong

**Affiliations:** ^1^ School of Pharmaceutical Sciences, Changchun University of Chinese Medicine, Changchun, 130117, Jilin, China, ccucm.edu.cn; ^2^ Jilin Ginseng Academy, Changchun University of Chinese Medicine, Changchun, 130117, Jilin, China, ccucm.edu.cn

**Keywords:** acute pneumonia, GEO data mining, liquid chromatography-mass spectrometry, metabolomics, zhuye shigao decoction

## Abstract

Zhuye Shigao Decoction (ZSD), a classic traditional Chinese medicine (TCM) formula, has demonstrated therapeutic efficacy in clinical settings for acute pneumonia; however, its mechanism of action remains elusive. This study aims to investigate the therapeutic effects and potential mechanisms of ZSD in lipopolysaccharide (LPS)‐induced acute pneumonia in mice. A liquid chromatography‐mass spectrometry (LC‐MS)‐based metabolomics approach, integrated with a gene expression omnibus (GEO) data mining technique, was employed in this investigation. The results indicated that ZSD administration significantly alleviated LPS‐induced pathological changes in lung tissue. In the model group, the concentrations of interleukin‐1*β* (IL‐1*β*), interleukin‐6 (IL‐6), and tumor necrosis factor‐*α* (TNF‐*α*) were increased, whereas the levels of immunoglobulin A (IgA) and immunoglobulin M (IgM) were significantly decreased. These indicators were reversed considerably in the ZSD‐high dose (ZSD‐H) group. A total of 118 metabolites exhibiting significant alterations in lung tissue were identified through metabolomics analysis. Following ZSD treatment, 84 of these metabolites were negatively regulated, and the associated metabolic processes implicated multiple pathways, including sphingolipid and arachidonic acid (AA) metabolism. Differentially expressed genes (DEGs) were identified through a comprehensive analysis of GEO datasets. Integrative pathway analysis identified signaling pathways associated with ZSD treatment effects in acute pneumonia. Notably, the Janus kinase/signal transducer and activator of transcription (JAK‐STAT) and Toll‐like receptor (TLR) signaling pathways emerged as critical contributors to these effects. This study indicates that ZSD can exert therapeutic effects on LPS‐induced acute pneumonia through a regulatory mechanism involving multiple components, targets, and pathways.

## 1. Introduction

Acute pneumonia represents a prevalent and life‐threatening respiratory condition recognized as one of the leading infectious causes of mortality on a global scale. This disease is characterized by its high incidence rates and significant contribution to both short‐term and long‐term mortality across all age demographics worldwide [1, 2]. Acute pneumonia commonly manifests with persistent cough, fever, dyspnea, and chest pain. Patients may also experience associated discomfort, including fatigue, anorexia, and increased sputum production. The diversity of pathogens combined with individual differences in the immune system makes the diagnosis and treatment of acute pneumonia a significant challenge in public health. Currently, drugs for acute pneumonia mainly include antibiotics and antiviral drugs, which can effectively inhibit the proliferation of pathogens and alleviate the symptoms of patients.

However, despite the widespread clinical use of such drugs, their limitations cannot be overlooked. First, the long‐term use of antibiotics can increase bacterial resistance, making subsequent treatments more complex. For example, acute pneumonia caused by Gram‐negative bacteria (GNB) has antibiotic‐resistant characteristics, making it difficult to treat [3–5]. Second, many drugs can cause specific side effects, affecting the life quality of patients. Consequently, relying on a single treatment approach proves insufficient for achieving complete efficacy. Hence, identifying drugs that combine high therapeutic effectiveness with minimal side effects is of great significance in the management of acute pneumonia.

In recent years, the efficacy and safety of traditional Chinese medicine (TCM) have gained widespread recognition, positioning it as a vital component in healthcare by the public, governmental bodies, and the World Health Organization (WHO) [6]. Particularly, in the treatment of respiratory diseases, TCM adopts a holistic approach that not only addresses symptoms but also considers the overall health of patients, emphasizing personalized treatment strategies. This approach has proven pivotal in mitigating the impact of the COVID‐19 pandemic [7]. Zhuye Shigao Decoction (ZSD) is a classic TCM formula used to treat diseases caused by heat pathogens, especially inflammation‐related symptoms [8]. ZSD has been considered an effective method to regulate lung health with its characteristics of clearing away heat, detoxifying, and relieving lung and cough. Its therapeutic effects have also been confirmed in modern clinical studies, but the specific mechanism of action is still unclear [9]. Given the inherent complexity of the TCM system, it is imperative to employ robust and advanced scientific methodologies to elucidate the mechanisms underlying the efficacy of ZSD in the treatment of acute pneumonia, which will ensure that ZSD can be safer and more rationally integrated into the modern medical system.

Several strategies have been developed to uncover the underlying mechanisms hidden behind the efficacy of TCM [10–13]. Metabolomics has shown extensive application potential in disease mechanism research, drug development, and personalized medicine, especially in the field of TCM research [14]. Metabolomics reveals body changes in different physiological and pathological states by a comprehensive analysis of metabolites in organisms. This attribute aligns with the holistic perspective of TCM, which treats illnesses by fostering balance within the human body and life. As a systematic approach, metabolomics provides an opportunity to elucidate the mechanism of action of TCM, TCM syndrome, and TCM treatment efficacy [15–17]. Gene expression omnibus (GEO) serves as a comprehensive repository, consolidating a vast array of gene expression datasets generated from diverse experimental designs. This publicly accessible platform provides an invaluable resource for scientific research. By enabling the exploration of disease mechanisms from multiple perspectives, GEO facilitates the identification of disease‐associated genes and pathways [18]. Furthermore, through the integration and systematic analysis of these datasets, researchers can generate novel hypotheses and uncover new insights into biological processes.

This study aims to explore the therapeutic effect of ZSD on an acute pneumonia model induced by lipopolysaccharide (LPS) and to analyze its mechanism of action. Inflammatory markers and lung tissues were examined to ascertain the drug efficacy. Liquid chromatography‐mass spectrometry (LC‐MS)‐based metabolomics technology was employed to monitor alterations in lung tissue metabolites. Subsequently, statistically significant metabolites were screened as biomarkers, and their related metabolic pathways were elucidated. Combined with GEO data mining, the antiinflammatory effect of ZSD and its potential application in acute pneumonia were comprehensively studied. This study anticipates generating novel perspectives on the integration of TCM within contemporary healthcare frameworks and furnishing substantial evidence for the management of acute pneumonia.

## 2. Materials and Methods

### 2.1. Chemicals and Reagents

HPLC acetonitrile, methanol, and isopropanol were purchased from Fisher Scientific Corporation (Waltham, USA). Formic acid and dichloromethane were purchased from Aladdin Industrial Corporation (Shanghai, China). Pure water was bought from Watsons (Guangzhou, China). Dexamethasone acetate tablets were purchased from Suicheng Pharmaceutical Co., Ltd., with product batch number 2306152. The ELISA kit (202,407) was purchased from Shanghai Elite Biotechnology Co., Ltd. (Shanghai, China). LPS (N28IS220883) was purchased from Shanghai Yuanye Biotechnology Co., Ltd. (Shanghai, China). All the herbs in Table S1 were provided by Hebei Shenwei Pharmaceutical Co., Ltd. (Shijiazhuang, China).

### 2.2. Preparation of ZSD

The ZSD was prepared based on the preliminary experimental investigations conducted in the laboratory [19]. The herbal formulation, comprising 27.6 g of Zhuye, 27.7 g of Renshen, 34.5 g of Banxia, 106 g of Maidong, 220 g of Shigao, and 27.6 g of Gancao, was prepared by placing the ingredients in a ceramic pot, adding 2 L of water, and allowing the mixture to soak for 30 min. The mixture was brought to a boil over 15 min using an electric ceramic stove with a setting of 2200 W. Subsequently, the heat was reduced to 400 W, and the decoction process was continued for an additional 30 min until the volume was reduced to 1.2 L. The resulting decoction was then filtered through a 200‐mesh gauze. 110.40 g of Jingmi was incorporated into the filtrate, boiled, and subsequently filtered again. The rice was cooked until fully gelatinized, then strained. The final volume was adjusted to 0.6 L and filtered again through a 200‐mesh gauze. Finally, the filtrate was freeze‐dried, pulverized, and homogenized to obtain the ZSD reference sample.

### 2.3. Animals and Treatment

The animal treatment was conducted as described in our previous study [20]. Fifty healthy ICR male mice weighing 33–37 g were provided by Changchun Yisi Animal Co., Ltd. (Changchun, China). The animal experiment was approved by the Animal Ethics Committee of Changchun University of Traditional Chinese Medicine (approval number: 2,024,313). All animal experiments followed the National Standard of the People’s Republic of China on Experimental Animals and Welfare (GB/T 42,011‐2022). All mice were maintained in a climate‐controlled environment with a temperature of 20–25°C, relative humidity of 40%–55%, and a light–dark cycle of 12 h. After a 7‐day adaptation period, the mice were randomly divided into five groups, each consisting of 10 mice: the control group, model group, positive group, ZSD‐low dose group (ZSD‐L), and ZSD‐high dose group (ZSD‐H). Anesthetized mice were positioned at a 45° angle, the oral cavity was opened, and the tongue was gently extended. Subsequently, 50 μL of LPS solution (1 mg/mL) was slowly instilled into the posterior pharyngeal wall using a micropipette. Immediately following instillation, the nares were temporarily occluded, and after 30 s, both the tongue and nares were released. The control group was given an equal volume of saline in the same way. After two consecutive days of modeling, except for the Control and Model groups, which were given equal amounts of saline, the remaining groups were administered the following doses for 1 week: Positive group (dexamethasone, 0.6 mg/kg, i.g.), ZSD‐L group (ZSD, 5.2 g/kg, i.g.), and ZSD‐H group (ZSD, 10.4 g/kg, i.g.).

### 2.4. Biochemical Analysis

The levels of immunoglobulin M (IgM), immunoglobulin A (IgA), IL‐1*β*, IL‐6, and TNF‐*α* in the lung tissues of mice were detected by ELISA assay. The left lung lobes of mice in each group were stained with hematoxylin and eosin (H&E) to observe the morphological changes in lung tissues.

### 2.5. Metabolomics Analysis

#### 2.5.1. Preparation of Lung Tissue Sample

About 50 mg of lung tissue samples were taken from each group separately and homogenized by adding 1.5 mL of cold methanol/water (1:1). The sample was centrifuged at 12,000 g for 10 min at 4°C. Following centrifugation, the supernatant, representing the aqueous phase, was carefully aliquoted. The resulting pellet, containing the sedimented tissue, was retained for subsequent organic‐phase extraction. The collected supernatant was then dried under a gentle stream of nitrogen and subsequently reconstituted in 100 μL of methanol/water (1:1, v/v). The resulting solution was vortexed for 30 s and centrifuged at 12,000 g for 15 min at 4°C. Finally, 5 μL of the supernatant was carefully removed and subjected to LC‐MS analysis. A quality control (QC) sample was prepared by pooling 10 μL aliquots from each sample. This QC sample was used for the method validation of LC‐MS conditions.

The tissue sediment was immediately placed on ice and homogenized in 1.6 mL of ice‐cold dichloromethane/methanol (3:1, v/v). The homogenate was centrifuged at 10,000 g for 10 min at 4°C, and the resulting supernatant, representing the organic phase, was collected. The collected supernatant was then evaporated to dryness under a stream of nitrogen, reconstituted in 100 μL of methanol/water (1:1, v/v), and vortexed for 30 s. Following centrifugation at 15,000 g for 15 min at 4°C, 80 μL of the resulting supernatant was carefully removed and subjected to LC‐MS analysis. The QC sample was prepared by pooling 10 μL aliquots from each sample.

#### 2.5.2. UHPLC‐MS Analysis

Metabolic profiling of lung tissue samples was analyzed using an ultra‐high‐performance liquid chromatography‐quadrupole time of flight mass spectrometer (UHPLC‐Q‐TOF MS). Sample separation was performed on an ACQUITY BEH C18 column (50 mm × 2.1 mm, particle size: 1.7 μm) with a column temperature of 50°C. The mobile phase comprised 0.1% formic acid in water (Phase A) and acetonitrile (Phase B) and was delivered at a flow rate of 0.4 mL/min under the following gradient conditions: 3%–70% B (1–8 min); 70% B (8–10 min); 70%–90% B (10–17 min); 90%–100% B (17–18 min); 100% B (18–21 min); 100%–3% B (21–22 min); and 3% B (22–27 min). The injected volume was 5 μL.

After chromatographic separation, the eluate was injected into the SYNAPT G2Si Q‐TOF MS (Waters Corp., Manchester, UK) with an electrospray ionization (ESI) source. Data acquisition was conducted in both positive and negative ionization modes. Full‐scan analysis was performed over a m/z range of 100–1500 in resolution mode. Tandem mass spectrometry data were acquired using a fast data‐dependent acquisition (fast‐DDA) method, selecting the top 15 precursor ions for fragmentation. The ionization source parameters were optimized as follows: capillary voltage, 3.0 kV or –2.5 kV; source temperature, 150°C; sample cone voltage, 40 V; desolvation temperature, 500°C; nitrogen gas flow, 900 L/h; and cone gas flow, 50 L/h. The mass spectrometer was calibrated with a 0.5 mM sodium formate solution to ensure mass accuracy prior to data acquisition. The real‐time calibration was performed during sample analysis using a leucine‐enkephalin solution (200 ng/mL).

#### 2.5.3. Data Processing and Identification of Metabolite Selection

Before statistical analyses, raw data were transformed into structured datasets using Progenesis QI software (Nonlinear Dynamics, Newcastle, UK). LC‐MS data from lung tissue samples underwent preprocessing, which included peak extraction, peak alignment, and peak intensity normalization. Subsequently, a comprehensive dataset was generated, comprising sample codes, retention times (RT), m/z, and peak intensities for subsequent data analysis. This dataset was then imported into SIMCA software (version 14.1, Umetrics, Umeå, Sweden) for multivariate statistical analysis. Partial least squares discriminant analysis (PLS‐DA) models were constructed based on the generated dataset for pattern recognition. The performance of the PLS‐DA model was evaluated using the parameters R2Y and Q2 (cum). Features were initially filtered based on variable importance in projection (VIP) scores. Subsequently, *t*‐tests and fold change (FC) analyses were performed to assess significant differences in these features and to identify potential biomarkers.

Metabolites in lung tissue were identified by matching high‐resolution mass spectrometry (HRMS) data with entries in the public Human Metabolome Database (HMDB) (https://www.hmdb.ca). Metabolic pathway analysis of the identified biomarkers was then conducted using the MetaboAnalyst 6.0 web server (https://www.metaboanalyst.ca).

#### 2.5.4. GEO Data Mining

GEO (https://www.ncbi.nlm.nih.gov/geo/) is an international public database that stores high‐throughput microarray and next‐generation genomic data. We searched the GEO database using the keyword “acute pneumonia” and selected the LPS‐induced mouse model dataset (GSE2411, GSE18341, and GSE48787) for analysis. Differentially expressed genes (DEGs) were screened according to *p*  < 0.05 and FC > 1.5. A Venn diagram was utilized to select the overlapping DEGs across the three datasets. Combining with metabolomics data, we conducted a joint pathway analysis to enrich significant signaling pathways by importing metabolic biomarkers and regulated DEGs into the MetaboAnalyst web platform.

## 3. Results

### 3.1. Effect of ZSD on LPS‐Induced Acute Pneumonia

**Figure 1:**
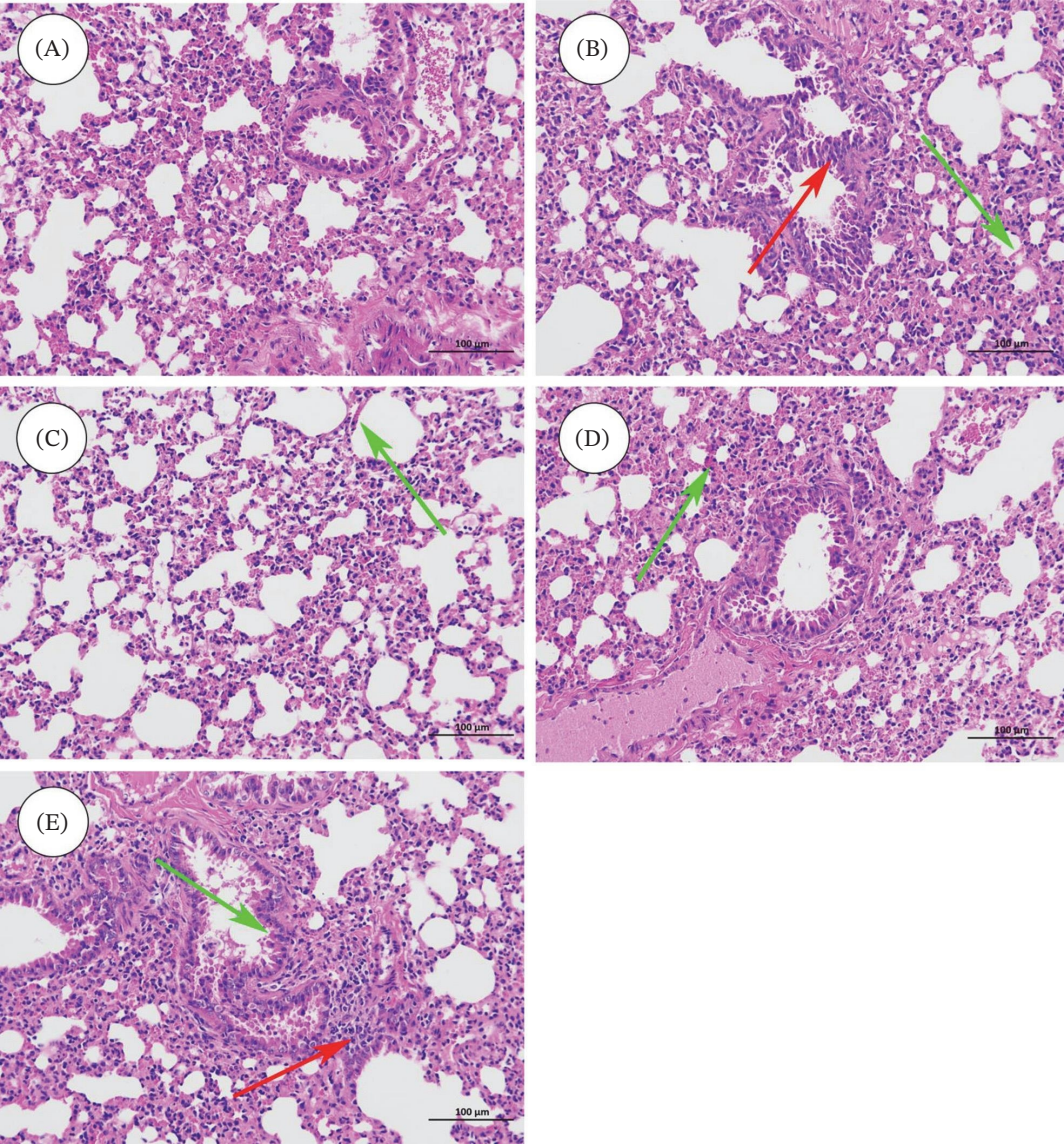
HE‐stained pathological sections of lung tissue of control (A), model (B), positive (C), ZSD‐H (D), and ZSD‐L (E) groups. Red arrows indicate inflammatory cell aggregation. Green arrows indicate airway wall thickening.

H&E staining of lung tissue sections was performed to observe morphological changes and to assess the effect of ZSD on inflammatory lesions, and the results are shown in Figure 1. In the control group, the bronchial mucosal epithelium and airway smooth muscle layer of the lung tissue remain intact, and the bronchial lumen is regularly shaped with less inflammatory cell infiltration. However, in LPS‐induced lung tissues, infiltration of inflammatory cells into perivascular and connective tissues, lumen narrowing, and mucosal thickening are observed. Dexamethasone and ZSD treatments significantly alleviate the pathologic changes induced by LPS. These results suggest that ZSD has a significant therapeutic effect on LPS‐induced acute pneumonia.

**Figure 2:**
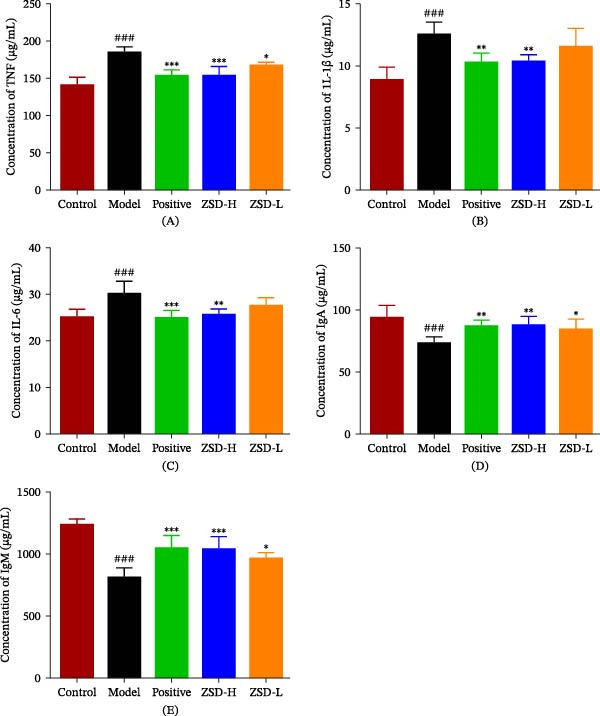
The expression of inflammatory factors and immune proteins in the lung tissue of each group of mice. Compared with the control group, ^###^ *p* < 0.001; compared with the model group,  ^∗^ *p*＜0.05,  ^∗∗^ *p*＜0.01,  ^∗∗∗^ *p*＜0.001.

As shown in Figure S1A, the loss of body weight is observed during the LPS‐induced modeling, but these losses are alleviated after drug treatment. Compared with the control group, the lung index in the model group significantly increases (Figure S1B), while the drug treatment dramatically reduces this increased index. These results suggest that the ZSD treatment has a certain therapeutic effect on the abnormalities of the physiological conditions of mice induced by LPS and can relieve the degree of pulmonary edema in LPS‐induced acute pneumonia mice. Upon introduction into the pulmonary system, LPS stimulates immune cells to release inflammatory cytokines, thereby eliciting an inflammatory response. Although the inflammatory response helps clear LPS and other pathogens, excessive inflammation may cause damage to lung tissue [21, 22]. The antiinflammatory effect of ZSD on LPS‐induced acute pneumonia was assessed by detecting the IL‐1*β*, IL‐6, and TNF‐*α* levels in lung tissues. As depicted in Figure 2A–C, the model group exhibits markedly elevated concentrations of IL‐1*β*, IL‐6, and TNF‐*α* compared to the control group; however, ZSD‐H treatment results in a significant reduction in these cytokine levels. IgA is an essential component of the respiratory mucosal barrier, and IgM is the earliest immunoglobulin to appear. IgA and IgM are primary antibodies in the body’s immunity against infections, and immunoglobulin levels correlate with immune function [23]. Pulmonary IgA and IgM concentrations are significantly reduced in the model group compared to the control group (Figure 2D,E), indicating immune dysfunction in model mice. However, following treatment administration, a significant regression is observed across all measured immune indices, with the ZSD‐H group exhibiting the most pronounced effect.

### 3.2. LC‐MS Method Validation

The repeatability and stability of the LC‐MS method were evaluated through replicate injections of QC samples. The first 5 QC sample injections were used to maintain system equilibrium, followed by 1 QC sample injection at 6 intervals to validate the method. After raw data preprocessing, two datasets were created for aqueous and organic extracts. The relative standard deviation (RSD%) of the ion signals in QC injections was calculated to validate the analytical method. The RSD% of ions in the aqueous and organic extracts is mainly below 30%, indicating that the established LC‐MS method possesses good reproducibility and stability.

### 3.3. Metabolome Profile and Pattern Recognition of Lung Tissue

**Figure 3:**
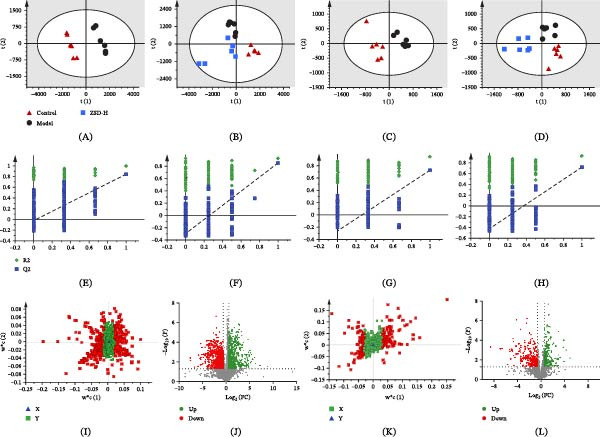
PLS‐DA score plots (A–D), permutation test (E–H), loading plots (I, K), and volcano plots (J, L) of control, model, and ZSD‐H groups in aqueous‐phase extracts based on the data from positive (A, B, E, F, I, J) and negative (C, D, G, H, K, L) ion model.

**Figure 4:**
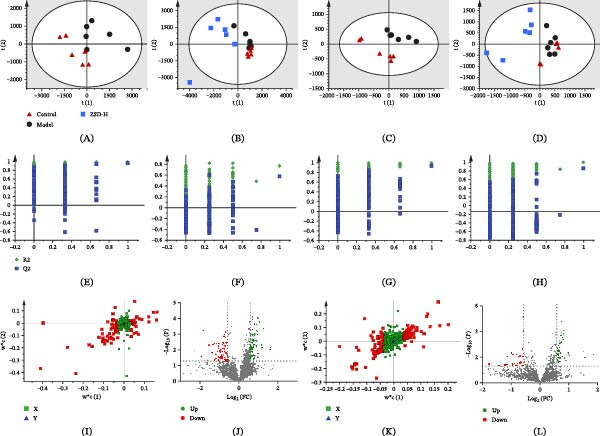
PLS‐DA score plots (A–D), permutation test (E–H), loading plots (I, K), and volcano plots (J, L) of control, model, and ZSD‐H groups in organic‐phase extracts based on the data from positive (A, B, E, F, I, J) and negative (C, D, G, H, K, L) ion model data.

Metabolites in lung tissue samples were analyzed using the validated LC‐MS method, and typical base peak chromatograms (BPC) are shown in Figures S2 and S3. Under this data collection condition, metabolites are well‐separated in 25 min, and differences are observed between populations. Changes in lung tissue metabolites across groups were analyzed using statistical methods to further assess the efficacy of ZSD. A PLS‐DA model was developed to elucidate the distribution of lung tissue samples within each group and to visualize the inherent classification patterns. The R2Y and Q2 (cum) values of the data for the aqueous and organic extract are shown in Table S2, indicating good adaptability and predictability of the model. For the aqueous metabolite, the score plots show that the control and model groups are clearly separated in both positive and negative ion modes, suggesting that the concentration and types are altered by the LPS intervention (Figure 3A,C). After ZSD treatment, the samples of the ZSD‐H group are separated from the model group and are closer to the control group, suggesting that ZSD partially alters metabolic changes and exerts therapeutic effects in LPS‐induced acute pneumonia mice **(**Figure 3B,D). The permutation test (*n* = 200) shows that the Q2 and R2 values are lower than the original value, indicating no overfitting in established models (Figure 3E–H). Similar results are also observed in the analysis of organic extract (Figure 4A–H).

### 3.4. Selection of Biomarkers and Pathway Enrichment Analysis

Several parameters, including VIP scores, FC, and *p*‐values, were employed to identify the metabolites that most significantly contributed to the discrimination between samples from the control and model groups. Initially, variables with VIP scores exceeding 1.0 were selected, followed by the application of FC > 1.5 and *t*‐tests (*p*  < 0.05) to further refine the selection of statistically significant metabolites (Figures 3I–L and 4I–L). The accurate mass spectrometry and tandem mass spectrometry information were used for metabolite annotation through database searches.

**Figure 5:**
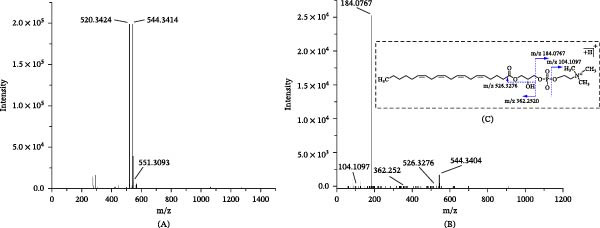
MS (A) and MS/MS (B) spectra of LysoPC (20:4/0:0) obtained in positive ion mode and the possible cleavage pathway of LysoPC (20:4/0:0) (C).

**Figure 6:**
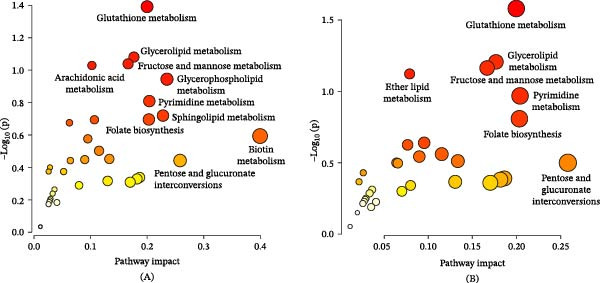
Metabolic pathways regulated by ZSD in LPS‐induced acute pneumonia in aqueous‐phase extracts (A) and organic‐phase extracts (B).

The metabolite annotation procedure was elucidated using the compound LysoPC (20:4/0:0) as an example, which has an exact molecular weight of 543.3337 at 6.48 min. As shown in Figure 5A, the protonated ion at m/z 544.3414 is the base peak of the spectrum. The tandem MS spectrum in Figure 5B shows that the fragment ions observed at m/z 526.3276 and m/z 104.1097 correspond to [M‐H_2_O+H]+ and [(CH_3_)3N(CH_2_)2O+H]+ of the head group, respectively. The fragment ions at m/z 184.0767 and m/z 362.2520 correspond to [PC headgroup+H]+ and [M‐PC headgroup+H]+, respectively. Based on the above information, the ion was assigned to LysoPC (20:4/0:0), and the possible cleavage pathway is shown in Figure 5C. Finally, 118 potential metabolic biomarkers associated with acute pneumonia were annotated (Tables 1 and 2). A comparison of metabolite intensities between the ZSD‐H group and the Model group showed significant changes in 84 out of 118 metabolites (*p*  < 0.05). Alterations in these metabolites indicate shifts in metabolic phenotypes, potentially elucidating the mechanisms underlying ZSD efficacy. Differential metabolites that ZSD reversely regulated were selected and subsequently subjected to pathway enrichment analysis using MetaboAnalyst. The results are presented in Figure 6A,B. The metabolic pathways modulated by ZSD in the context of LPS‐induced acute pneumonia include sphingolipid metabolism, arachidonic acid (AA) metabolism, glutathione metabolism, and glycerophospholipid metabolism.

**Table 1:** Aqueous extracted metabolic biomarkers of lung tissue associated with acute pneumonia and their trends in different groups.

No.	Metabolite	Retention time (min)	Chemical formula	Detected (m/z)	Mass error (ppm)	Adducts	HMDB ID	Model vs control	HD vs model
1	Tetranor 12‐HETE	6.54	C16H26O3	311.1876	4.44	[M+FA‐H]−	HMDB0060055	↓ ^∗∗^	↓
2	Prostaglandin B‐2	4.39	C20H30O4	317.2138	8.05	[M+H‐H_2_O]+	HMDB0302205	↑ ^∗^	↓#
3	12‐hydroxyicosanoic acid	6.66	C20H40O3	346.3341	7.79	[M+NH_4_]+	HMDB0061664	↓ ^∗∗^	↑
4	Prostaglandin G1	4.39	C20H34O6	393.2263	4.13	[M+Na]+	HMDB0013039	↑ ^∗^	↓#
5	Leukotriene E3	5.79	C23H39NO5S	442.2654	7.32	[M+H]+	HMDB0002355	↓ ^∗∗^	↓
6	*N*‐acetyl‐leukotriene E4	2.99	C25H39NO6S	516.2231	8.12	[M+Cl]−	HMDB0005084	↓ ^∗∗^	↑
7	Dehydrodolichol diphosphate	3.50	C25H44O7P2	519.2669	6.62	[M+H]+	HMDB0060469	↑ ^∗∗^	↓
8	LysoPC(0:0/18:2)	7.28	C26H50NO7P	520.3409	2.18	[M+H]+	HMDB0010386	↑ ^∗^	↓
9	LysoPC(0:0/20:4)	7.43	C28H50NO7P	544.3409	1.52	[M+H]+	HMDB0061699	↑ ^∗∗^	↓
10	PA(2:0/22:6‐2OH)	7.04	C27H41O10P	555.2381	2.93	[M‐H]−	HMDB0266553	↑ ^∗∗^	↓
11	LysoPS(18:2/0:0)	7.82	C24H44NO9P	566.2761	4.86	[M+FA‐H]−	HMDB0240604	↑ ^∗^	↓
12	LysoPS(18:1/0:0)	8.65	C24H46NO9P	568.2921	5.49	[M+FA‐H]−	HMDB0240603	↑ ^∗∗∗^	↑#
13	PC(2:0/20:3‐OH)	7.29	C30H54NO9P	568.3403	0.93	[M+H‐2H_2_O]+	HMDB0288900	↑ ^∗∗^	↓
14	LysoPI(16:0/0:0)	3.97	C25H49O12P	573.3002	−5.58	[M+H]+	HMDB0061695	↑ ^∗∗∗^	↓
15	18‐oxo‐oleate	4.62	C18H31O3	591.4591	−4.77	[2M+H]+	HMDB0304034	↑ ^∗∗^	↓##
16	Sphinganine	4.64	C18H39NO2	603.6068	8.98	[2M+H]+	HMDB0000269	↑ ^∗∗^	↓##
17	PA(8:0/20:5‐3OH)	3.84	C31H51O11P	613.3135	−0.12	[M+H‐H_2_O]+	HMDB0266657	↑ ^∗∗∗^	↓##
18	DG(15:0/22:1/0:0)	4.09	C40H76O5	637.5787	3.41	[M+H]+	HMDB0007087	↑ ^∗∗∗^	↓
19	Cer(d18:0/23:0)	2.77	C41H83NO3	660.6269	0.66	[M+Na]+	HMDB0011767	↑ ^∗∗^	↓#
20	DG(22:6‐OH/17:0/0:0)	3.76	C42H70O6	671.5294	7.28	[M+H]+	HMDB0295760	↑ ^∗∗^	↑##
21	Omega‐carboxy‐trinor‐leukotriene B4	2.84	C18H26O6	677.3469	−9.31	[2M+H]+	HMDB0013032	↑ ^∗∗∗^	↓
22	PE(14:1/16:1)	2.84	C35H66NO8P	677.4909	6.77	[M+NH_4_]+	HMDB0008858	↑ ^∗∗∗^	↓#
23	DG(PGJ2/i‐19:0/0:0)	4.62	C42H72O7	689.5342	−1.29	[M+H]+	HMDB0299828	↑ ^∗∗^	↓##
24	PA(15:0/20:2)	5.67	C38H71O8P	704.5195	−4.39	[M+NH_4_]+	HMDB0115508	↓ ^∗∗∗^	↑
25	CE(MonoMe(11,5))	4.48	C48H80O3	705.6163	−2.50	[M+H]+	HMDB0061673	↓ ^∗∗^	↑
26	MG(P‐18:0/0:0/0:0)	4.71	C21H42O3	707.6113	−6.88	[2M+Na]+	HMDB0011153	↓ ^∗^	↑
27	DG(20:0/22:6‐2OH/0:0)	2.72	C45H76O7	746.5914	−2.10	[M+NH_4_]+	HMDB0296367	↓ ^∗∗^	↓
28	PE(20:4‐OH/15:0)	5.63	C40H72NO9P	764.4872	4.71	[M+Na]+	HMDB0260791	↓ ^∗∗∗^	↑
29	PC(18:0/15:0)	2.77	C41H82NO8P	770.5619	−6.88	[M+Na]+	HMDB0008033	↑ ^∗∗^	↓
30	PA(LTE4/12:0)	5.32	C38H66NO11PS	776.4231	8.27	[M+H]+	HMDB0262781	↑ ^∗∗^	↑
31	SM(d16:1/20:3‐2OH(5,6))	5.28	C41H77N2O8P	779.5283	−3.47	[M+Na]+	HMDB0290273	↓ ^∗∗^	↑
32	PA(24:0/20:1)	4.53	C47H91O8P	779.6304	−1.08	[M+H‐2H_2_O]+	HMDB0115441	↓ ^∗∗∗^	↑
33	PC(14:1/20:0)	2.78	C42H82NO8P	782.5676	0.75	[M+Na]+	HMDB0007911	↑ ^∗∗∗^	↓##
34	PG(i‐12:0/PGE2)	4.25	C38H67O13P	785.4151	−7.89	[M+Na]+	HMDB0271073	↑ ^∗∗^	↑
35	PA(18:1/24:0)	5.65	C45H87O8P	787.6235	2.96	[M+H]+	HMDB0114919	↓ ^∗∗∗^	↑
36	PC(18:0/20:5))	2.84	C46H82NO8P	790.5691	−6.70	[M+H‐H_2_O]+	HMDB0008050	↑ ^∗∗∗^	↓#
37	TG(18:3/14:0/O‐18:0)	5.72	C53H98O5	815.7535	5.85	[M+H]+	HMDB0055154	↓ ^∗∗∗^	↑
38	PS(14:0/20:5‐3OH)	5.68	C40H68NO13P	819.4716	−6.30	[M+NH_4_]+	HMDB0280869	↓ ^∗∗∗^	↑
39	Oleylcarnitine	0.31	C25H49NO3	821.7348	−0.46	[2M‐H]‐	HMDB0255949	↓ ^∗^	↑
40	PE(20:5‐3OH/DiMe)	5.11	C43H70NO12P	824.4724	1.91	[M+H]+	HMDB0285033	↓ ^∗∗∗^	↓
41	PS(14:0/PGF1*α*)	5.77	C40H74NO13P	830.4748	−5.22	[M+Na]+	HMDB0280895	↑ ^∗^	↓#
42	PA(20:5‐3OH/21:0)	4.03	C44H77O11P	830.5568	3.20	[M+NH_4_]+	HMDB0265518	↑ ^∗∗∗^	↓###
43	TG(a‐21:0/a‐13:0/i‐16:0)	4.70	C53H102O6	835.7745	−0.50	[M+H]+	HMDB0110818	↑ ^∗∗∗^	↓
44	PS(TXB2/14:1)	4.69	C40H70NO14P	842.4440	1.74	[M+Na]+	HMDB0280941	↑ ^∗∗^	↓##
45	PG(18:1/PGJ2)	5.62	C44H75O12P	844.5324	−1.28	[M+NH_4_]+	HMDB0269003	↓ ^∗∗∗^	↑
46	PA(PGE1/22:2)	2.78	C45H79O11P	844.5764	7.91	[M+NH_4_]+	HMDB0265851	↓ ^∗∗∗^	↑
47	dADP	4.64	C10H15N5O9P2	845.0508	−9.00	[2M+Na]+	HMDB0001508	↑ ^∗∗^	↓
48	PI(16:2/18:0)	5.06	C43H79O13P	857.5159	0.99	[M+Na]+	HMDB0009803	↓ ^∗∗∗^	↑
49	SM(d20:1/22:6‐2OH)	3.91	C47H83N2O8P	857.5724	−6.67	[M+Na]+	HMDB0290754	↓ ^∗∗∗^	↑
50	PGP(PGE2/i‐12:0)	3.98	C38H68O16P2	860.4381	7.10	[M+NH_4_]+	HMDB0274810	↓ ^∗∗∗^	↑
51	PG(16:1/PGE2)	21.76	C42H73O13P	861.4736	−4.28	[M+FA‐H]−	HMDB0268683	↓ ^∗∗^	↓
52	PC(20:0/20:3‐2OH)	5.64	C48H90NO10P	872.6343	−3.64	[M+H]+	HMDB0286976	↓ ^∗∗∗^	↑
53	PGP(20:4/22:6)	4.52	C48H76O13P2	887.4590	−3.58	[M+H‐2H_2_O]+	HMDB0116488	↑ ^∗^	↓
54	PC(DiMe(11,5)/22:6‐OH)	5.66	C52H86NO10P	898.5912	−4.83	[M+H‐H_2_O]+	HMDB0289086	↓ ^∗∗∗^	↑
55	5‐Decenoyl‐CoA	4.63	C31H52N7O17P3S	902.2325	0.46	[M+H‐H_2_O]+	HMDB0300870	↓ ^∗∗∗^	↓
56	PC(22:5/24:0)	5.63	C54H98NO8P	902.7078	8.74	[M+H‐H_2_O]+	HMDB0008683	↓ ^∗∗∗^	↑
57	PG(22:5/20:5‐3OH)	7.61	C48H75O13P	913.4762	−8.48	[M+Na]+	HMDB0270288	↑ ^∗^	↓
58	PE(TXB2/DiMe)	5.30	C47H82NO13P	917.5873	1.23	[M+NH_4_]+	HMDB0284793	↑ ^∗∗^	↑
59	PGP(20:3‐OH(5)/a‐21:0)	4.43	C47H88O14P2	921.5632	1.66	[M+H‐H_2_O]+	HMDB0274676	↑ ^∗∗∗^	↓#
60	20‐Oxo‐leukotriene E4	2.77	C23H35NO6S	924.4712	0.43	[2M+NH_4_]+	HMDB0012642	↑ ^∗∗^	↓
61	PS(22:4/24:0)	2.78	C52H94NO10P	924.6731	4.62	[M+H]+	HMDB0112813	↑ ^∗∗^	↓
62	*N*‐hexacosanoylglycine	2.78	C28H55NO3	924.8729	2.94	[2M+NH_4_]+	HMDB0062678	↑ ^∗∗^	↓
63	PGP(i‐16:0/PGD2)	21.76	C42H76O16P2	943.4550	−4.55	[M+FA‐H]−	HMDB0275227	↓ ^∗∗^	↓
64	PG(LTE4/18:3)	2.78	C47H78NO13PS	950.4839	1.68	[M+Na]+	HMDB0269318	↑ ^∗∗^	↓#
65	PS(22:2/24:0)	2.78	C52H98NO10P	950.6827	0.67	[M+Na]+	HMDB0112786	↑ ^∗∗^	↓#
66	PC(22:6‐2OH/22:3)	4.99	C53H88NO10P	952.6106	7.26	[M+Na]+	HMDB0288143	↓ ^∗∗∗^	↑
67	TG(18:1/24:0/O‐18:0)	4.65	C63H122O5	959.9371	0.60	[M+H]+	HMDB0049218	↑ ^∗∗^	↑
68	PC(22:1/TXB2)	0.31	C50H92NO12P	974.6261	−8.36	[M+FA‐H]−	HMDB0287905	↓ ^∗^	↓
69	DG(8:0/19:0/0:0)	4.64	C30H58O5	1014.8834	−7.33	[2M+NH_4_]+	HMDB0092937	↓ ^∗∗∗^	↓
70	PIP(PGE1/20:3)	7.28	C49H84O19P2	1019.4918	1.39	[M‐H_2_O‐H]−	HMDB0279857	↑ ^∗∗^	↓
71	Pentadecanedioyl‐CoA	5.63	C36H62N7O19P3S	1039.3271	−9.90	[M+NH_4_]+	HMDB0301243	↓ ^∗∗∗^	↑
72	Docosadienoyl‐CoA	4.68	C43H74N7O17P3S	1130.4035	−1.99	[M+FA‐H]‐	HMDB0062220	↓ ^∗^	↓
73	PIP(TXB2/22:3)	7.30	C52H90O20P2	1131.5303	9.87	[M+Cl]‐	HMDB0280322	↑ ^∗^	↓
74	CL(8:0/10:0/10:0/i‐19:0)	5.67	C56H108O17P2	1132.7479	7.12	[M+NH_4_]+	HMDB0117448	↓ ^∗∗∗^	↑
75	Biotinyl‐5^′^‐AMP	4.65	C20H28N7O9PS	1169.2720	1.26	[2M+Na]+	HMDB0004220	↓ ^∗^	↓
76	DG(15:0/20:1/0:0)	5.84	C38H72O5	1218.0798	−2.79	[2M+H]+	HMDB0007079	↑ ^∗∗^	↓#
77	DG(20:5‐OH/16:0/0:0)	4.69	C39H66O6	1259.9651	0.38	[2 M‐H]−	HMDB0295532	↑ ^∗∗∗^	↓##
78	CL(15:0/15:0/i‐16:0/i‐19:0)	4.52	C74H144O17P2	1347.9721	1.51	[M‐H_2_O‐H]−	HMDB0229800	↑ ^∗∗^	↓
79	CL(16:0/16:0/16:0/18:1)	4.52	C75H144O17P2	1359.9795	6.87	[M‐H_2_O‐H]‐	HMDB0056390	↑ ^∗∗^	↓
80	DG(17:0/0:0/20:5‐3OH	5.66	C40H68O8	1371.0156	−0.76	[2M+NH_4_]+	HMDB0295749	↓ ^∗∗^	↑
81	DG(19:0/0:0/22:6‐2OH)	5.68	C44H74O7	1447.1184	−1.63	[2M+NH_4_]+	HMDB0296161	↓ ^∗∗^	↑

*Note:* ^∗^ *p*  < 0.05,  ^∗∗^ *p*  < 0.01,  ^∗∗∗^ *p*  < 0.001, the model group versus the control group. ^#^ *p*  < 0.05, ^##^ *p*  < 0.01, ^###^ *p*  < 0.001, the ZSD‐H treatment group versus the model group.

**Table 2:** Organic extracted metabolic biomarkers of lung tissue associated with acute pneumonia and their trends in different groups.

No.	Metabolite	Retention time (min)	Chemical formula	Detected (m/z)	Mass error (ppm)	Adducts	HMDB ID	Model vs control	HD vs model
1	Glycerophosphocholine	6.23	C8H20NO6P	240.1001	2.12	[M+H‐H_2_O]+	HMDB0000086	↓ ^∗∗^	↓
2	13‐HOTE	8.44	C18H30O3	259.2039	−5.86	[M+H‐2H_2_O]+	HMDB0010203	↓ ^∗^	↑
3	6‐Octadecenoic acid	11.06	C18H34O2	265.2537	3.90	[M+H‐H_2_O]+	HMDB0303337	↑ ^∗^	↓
4	Hexadecadienoic acid	6.59	C16H28O2	275.1994	4.86	[M+Na]+	HMDB0302694	↓ ^∗∗^	↑
5	Oleic acid	8.25	C18H34O2	300.2895	−0.56	[M+NH_4_]+	HMDB0000207	↓ ^∗∗^	↑
6	2‐Undecyl‐4(1H)‐quinolinone N‐oxide	10.97	C20H28NO2	313.2043	−1.26	[M‐H]−	HMDB0032995	↓ ^∗∗^	↑
7	*N*‐myristoyl lysine	11.08	C20H40N2O3	321.2923	6.37	[M+H‐2H_2_O]+	HMDB0242055	↑ ^∗∗∗^	↓#
8	Tricosanoic acid	8.36	C23H46O2	337.3451	−3.90	[M+H‐H_2_O]+	HMDB0001160	↑ ^∗^	↑
9	MG(0:0/18:1/0:0)	9.41	C21H40O4	339.2878	−4.37	[M+H‐H_2_O]+	HMDB0011536	↑ ^∗^	↓
10	2,3‐Dinor‐TXB1	13.48	C19H34O5	341.2300	−9.73	[M‐H]−	HMDB0245413	↓ ^∗∗^	↑
11	(9E)‐tridec‐9‐enedioylcarnitine	8.30	C20H35NO6	403.2807	1.04	[M+NH_4_]+	HMDB0241323	↑ ^∗∗^	↑
12	MG(0:0/22:6/0:0)	8.18	C25H38O4	403.2811	−7.82	[M+H]+	HMDB0011557	↑ ^∗∗∗^	↑
13	Oleoylcarnitine	7.17	C25H47NO4	426.3544	−7.92	[M+H]+	HMDB0005065	↑ ^∗^	↓
14	LysoPC(15:0/0:0)	6.07	C23H48NO7P	482.3206	−7.29	[M+H]+	HMDB0010381	↓ ^∗∗^	↓
15	LysoPC(16:0/0:0)	5.73	C24H50NO7P	518.3210	−1.35	[M+Na]+	HMDB0010382	↓ ^∗∗^	↓
16	LysoPC(18:3/0:0)	5.93	C26H48NO7P	518.3214	−5.30	[M+H]+	HMDB0010387	↓ ^∗∗∗^	↓#
17	LysoPC(18:2/0:0)	6.23	C26H50NO7P	520.3367	−5.84	[M+H]+	HMDB0061700	↓ ^∗∗∗^	↓
18	LysoPC(20:4/0:0)	6.48	C28H50NO7P	544.3367	−5.66	[M+H]+	HMDB0010395	↓ ^∗∗∗^	↓#
19	LysoPC(18:0/0:0)	6.71	C26H54NO7P	546.3497	−6.26	[M+Na]+	HMDB0010384	↓ ^∗∗^	↓
20	CGP71422	6.87	C29H31N7O2	554.2564	8.32	[M+FA‐H]−	HMDB0013864	↓ ^∗∗∗^	↓
21	LysoPC(20:2/0:0)	6.57	C28H54NO7P	570.3503	−4.87	[M+Na]+	HMDB0010392	↓ ^∗∗∗^	↓#
22	*N*‐nervonoyl tryptophan	6.57	C35H56N2O3	570.4601	−5.12	[M+NH_4_]+	HMDB0242101	↓ ^∗∗∗^	↓#
23	LysoPC(20:1/0:0)	7.37	C28H56NO7P	572.3662	−4.39	[M+Na]+	HMDB0010391	↓ ^∗∗∗^	↓
24	LysoPC(22:1/0:0)	8.31	C30H60NO7P	600.4006	1.09	[M+Na]+	HMDB0010399	↑ ^∗∗^	↓
25	LysoPI(18:2/0:0)	7.06	C27H49O12P	631.2611	−7.42	[M+Cl]−	HMDB0240597	↓ ^∗∗^	↑
26	Cibaric acid	11.05	C18H28O5	647.3819	2.87	2[M‐H]−	HMDB0038580	↑ ^∗∗^	↓
27	DG(20:5‐OH/0:0/a‐25:0)	18.36	C48H84O6	774.6681	9.90	[M+NH_4_]+	HMDB0298233	↓ ^∗∗^	↑
28	LysoPE(0:0/14:1)	18.36	C19H38NO7P	845.4683	−1.84	[2 M‐H]−	HMDB0011471	↑ ^∗^	↑
29	PE(PGF2alpha/22:6)	16.93	C47H76NO11P	906.5110	−3.29	[M+FA‐H]−	HMDB0284369	↑ ^∗^	↓
30	PGP(22:6–2OH/a‐17:0)	17.75	C45H78O15P2	919.4760	1.87	[M‐H]−	HMDB0274542	↑ ^∗∗^	↓
31	LysoPA(20:5/0:0)	4.95	C23H37O7P	930.4966	8.13	[2M+NH_4_]+	HMDB0114748	↓ ^∗^	↓#
32	PGP(TXB2/i‐21:0)	6.89	C47H88O17P2	987.5651	8.28	[M+H]+	HMDB0275757	↑ ^∗∗^	↓
33	PIP2(16:0/20:3)	6.42	C45H83O19P3	1001.4515	−4.71	[M‐H_2_O‐H]−	HMDB0010041	↑ ^∗∗^	↓#
34	PIP(18:3/18:1‐2OH)	6.88	C45H80O18P2	1015.4849	4.84	[M+FA‐H]−	HMDB0279299	↑ ^∗^	↓
35	CDP‐DG(i‐17:0/22:6‐OH)	6.23	C51H83N3O16P2	1100.5225	−0.57	[M+FA‐H]−	HMDB0293856	↓ ^∗^	↑
36	CDP‐DG(22:5‐O/i‐17:0)	6.45	C51H83N3O16P2	1100.5258	2.61	[M+FA‐H]−	HMDB0293865	↓ ^∗^	↓
37	CDP‐DG(22:5/PGE1)	6.47	C54H85N3O18P2	1124.5243	1.13	[M‐H]−	HMDB0292519	↓ ^∗∗^	↓

*Note:* ^∗^ *p*  < 0.05,  ^∗∗^ *p*  < 0.01,  ^∗∗∗^ *p*  < 0.001, the model group versus the control group. ^#^ *p*  < 0.05, ^##^ *p*  < 0.01, ^###^ *p*  < 0.001, the ZSD‐H treatment group versus the model group.

### 3.5. GEO Data Mining Analysis

**Figure 7:**
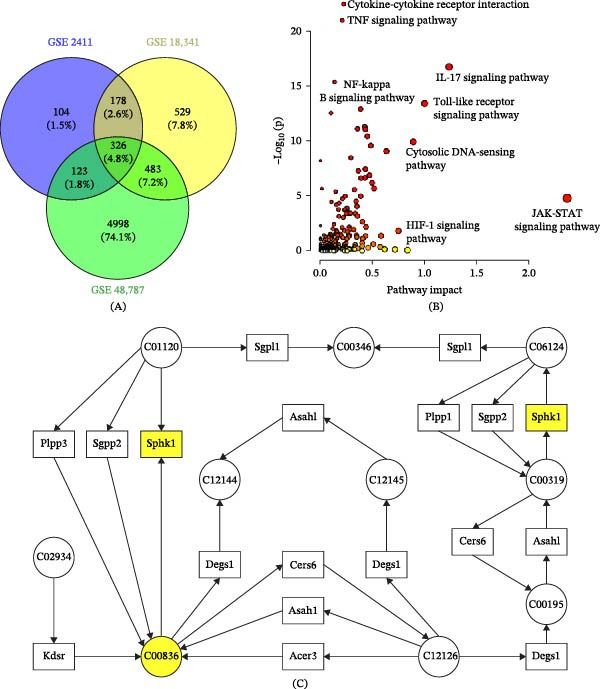
Venn diagram of DEGs selected from GSE2411, GSE18341, and GSE48787 (A). Enriched signaling pathways from joint pathway analysis (B). The representative pathways of sphingolipid metabolism (C). Circles represent metabolic biomarkers, squares represent DEGs, and yellow highlights the detected metabolites and genes.

The DEGs from three GEO datasets were selected, and 326 overlapping DEGs were highlighted using a Venn diagram (Figure 7A). Metabolic biomarkers and DEGs identified from the GEO database were subjected to integrated pathway analysis using MetaboAnalyst. The significantly enriched signaling pathways are presented in Figure 7B. The main pathways include the Janus kinase/signal transducer and activator of transcription (JAK‐STAT) signaling pathway, the Toll‐like receptor (TLR) signaling pathway, and the Cytokine–cytokine receptor interaction. Figure 7C depicts sphingolipid metabolism, demonstrating the regulation mechanism of ZSD in both metabolic and signaling pathways.

## 4. Discussion

Acute pneumonia is a respiratory disease caused by infection with a variety of pathogens. Even in the general case of a common cold or upper respiratory tract infection, it can rapidly progress to acute pneumonia if left untreated. Acute pneumonia has an abrupt onset, with symptoms such as chest tightness, cough, and fever. It has many complications, and in particularly severe cases, respiratory distress may occur. ZSD is a classic herbal formula traditionally known for its efficacy in clearing away heat and detoxifying, as well as moistening the lungs and relieving cough. It is clinically used to treat symptoms such as cough, pharyngitis, and fever. Furthermore, it is considered a therapeutic option for pulmonary inflammation; however, its precise mechanism of action remains to be fully elucidated.

LPS is a major component of GNB capable of triggering a strong inflammatory response. Recent epidemiological studies have demonstrated an elevated incidence of pneumonia attributed to GNB, potentially resulting in immune dysregulation and pulmonary tissue injury. GNB has also been investigated as an etiological agent in acute pneumonia [24]. In this study, we established a murine model of acute pneumonia via LPS induction and subsequently administered ZSD as a treatment. The etiology of acute pneumonia lies primarily in infection and inflammation of the lungs, so direct studies on lung tissue are more likely to provide accurate and reliable insights and data, which makes them more rational and practical. The levels of IgM, IgA, IL‐1*β*, IL‐6, and TNF‐*α* were quantified in lung tissue extracts. The results suggest that ZSD can enhance immune function while simultaneously attenuating the expression of inflammatory mediators. Histopathological examination of lung tissue via H&E staining reveals that, compared to the model group, the ZSD group exhibits reduced inflammatory cell infiltration and epithelial cell hyperplasia, indicating that ZSD can mitigate inflammatory responses and pathological damage, thereby demonstrating a potential therapeutic effect on acute pneumonia.

We investigated the antipneumonic activity of ZSD and its associated metabolism using LC‐MS‐based metabolomics. Following LPS‐induced stimulation, distinct segregation between the control and model groups is evident in the PLS‐DA score plots. The ZSD group tends to diverge from the model group, approaching the trajectory of the control group. A notable alteration in 37 lung tissue metabolites was observed in the organic phase extracts of the model group, with 19 exhibiting a return to baseline levels following ZSD treatment. In the aqueous‐phase extracts, 81 metabolites demonstrated significant changes by LPS, and ZSD treatment led to the normalization of 65 of these metabolites. These significantly reversed compounds are mainly sphinganine, phosphatidylcholine (PC), ceramide, AA, and prostaglandin (PG), which are involved primarily in sphingolipid metabolism, arachidonic acid metabolism, and glutathione metabolism. Integration of these metabolites with DEGs identified from the GEO database revealed a significant enrichment of potential signaling pathways implicated in the therapeutic mechanisms of ZSD, notably the TLR signaling pathway.

Sphingolipids are essential lipids characterized by a sphinganine backbone, which undergoes *N*‐acylation with diverse fatty acids, resulting in the formation of a wide array of ceramide species [25]. Studies have shown that ceramide accumulation can contribute to the inflammatory response through various mechanisms. For instance, the JAK‐STAT signaling pathway is implicated in the pathogenesis of inflammation. Many cytokines crucial to the pathogenesis of autoimmune and inflammatory diseases rely on JAK and STAT proteins to transduce intracellular signals. Notably, ceramide was shown to attenuate inflammatory airway diseases through the activation of the JAK2/STAT3 pathway within the airway epithelium [26, 27]. Elevated ceramide concentrations also stimulate macrophage activation and the subsequent release of proinflammatory mediators, thereby promoting macrophage infiltration into adipose tissue and instigating a localized inflammatory response.

Sphingolipid metabolism yields not only ceramide but also sphingosine‐1‐phosphate (S1P), a bioactive sphingolipid metabolite generated via the phosphorylation of sphingosine by sphingosine kinases (SphKs). There are two isoforms of sphingosine kinase: sphingosine kinase 1 (SphK1) and sphingosine kinase 2 (SphK2). S1P has been shown to induce proinflammatory signaling in the pulmonary vasculature by activating nuclear factor‐κB (NF‐κB) and the NLRP3 inflammasome, suggesting the involvement of the S1P/SphK1 signaling axis [28, 29]. SphK2 may also promote inflammation. The study demonstrated that SphK2 gene knockdown attenuated Gram‐negative bacteria‐induced lung inflammation, suggesting a potential therapeutic strategy for pneumonia [30]. In the present study, the enhancement of sphingosine and ceramide was observed in the model group compared to the control group, indicating that the sphingolipid metabolism was disturbed. The intensity of sphinganine and ceramide was downregulated after treatment with ZSD. The integrated pathway analysis showed that the expression of Sphk1 was changed by LPS administration (Figure 7C). The results suggest that ZSD possibly exerts a potential treatment effect on acute pneumonia by modulating the sphingolipid metabolism and the JAK‐STAT signaling pathway.

Eicosanoids are a class of biologically active lipids derived from AA, including PG, leukotriene (LT), thromboxane (TX), and lipoxins (LX). Excessive levels of many eicosanoids exhibit proinflammatory properties and participate in the metabolism of AA [31, 32]. The significant changes in leukotriene B4 (LTB4) and leukotriene E4 (LTE4) metabolites, including *N*‐acetyl‐LTE4 and omega‐carboxy‐trinor‐LTB4, were observed in the model group compared to the control group, whereas they were reversely regulated after ZSD administration. In a previous study, LTB4 and LTE4 levels were shown to be significantly elevated in bronchoalveolar lavage fluid from patients with lung disease and correlated with parameters of lung inflammation [33]. Besides, LTE4 was reported to play an important role in the proinflammatory response, increasing vascular permeability by attracting and activating mast cells, as well as enhancing the inflammatory response in the mucosa, a process that is particularly relevant to inflammation in the lung [34, 35].

PGs play essential roles in inflammation and immune responses. Prostaglandin G (PGG) is a precursor for PG biosynthesis, which is generated via the cyclooxygenase (COX) pathway and further converted to prostaglandin H (PGH), which ultimately generates the biologically active prostaglandin E (PGE) [36]. PGE has a dual role in the inflammatory response, facilitating the release of proinflammatory mediators while concurrently attenuating excessive inflammation via a negative feedback mechanism [37]. However, when PGG metabolism is dysregulated, it may lead to abnormal production of PGH and PGE, which in turn affects the balance of the inflammatory response. In the present study, PGG levels were significantly elevated in the model group, and the abnormal PGG metabolism may lead to insufficient PGE production, which weakened its antiinflammatory effect and exacerbated the response to acute pneumonia. PGB is a nonenzymatic degradation product of PGE, which is usually generated under acidic or oxidative stress conditions [38]. PGB was an inactive metabolite, but its accumulation may reflect enhanced oxidative stress and abnormal PGE metabolism. In this study, PGB levels were significantly higher in the model group, which was reported to be positively correlated with the oxidative stress marker PGF2*α*. The results suggest that the accumulation of PGB may stem from heightened oxidative stress and, furthermore, can exacerbate the pathological progression of acute pneumonia by attenuating the antiinflammatory and antioxidant properties of PGE. The levels of both PGG and PGB were significantly regressed under the therapeutic effect of ZSD, which further suggests that ZSD can regulate AA metabolism by affecting PGs and exert a therapeutic effect on acute pneumonia.

The integrative analysis of DEGs and metabolic biomarkers suggests a potentially significant role for TLR signaling pathways in the therapeutic effects of ZSD on acute pneumonia. TLRs play an essential role in the innate immune response and are capable of triggering both acute and chronic inflammatory responses, thus influencing the inflammatory process and its severity [39]. TLR4, the prototypic member of the TLR family, is a transmembrane protein critically involved in the innate immune response to pneumonia, especially in the setting of Gram‐negative bacterial infections. The LPS used in this study was derived from GNB. According to previous studies, TLR4 was capable of recognizing external pathogens by binding to the LPS of Gram‐negative bacteria and subsequently stimulating the production of antimicrobial peptides, which, in turn, could trigger a nonspecific immune response [24, 40]. TLR4 activation by LPS is mediated by the interaction of LPS with a cohort of proteins, notably lipopolysaccharide‐binding protein (LBP), myeloid differentiation protein 2 (MD2), and cluster of differentiation 14 (CD14), culminating in the formation of the LPS/MD2/TLR4 complex, which subsequently triggers intracellular signaling cascades [41]. AA has been demonstrated to interact with MD2, impeding MD2/TLR4 dimerization and modulating TLR4 activity via interactions with LPS and saturated fatty acids, ultimately culminating in inflammatory factor production and tissue damage [42]. These results suggest that ZSD can regulate the TLR signaling pathway and AA metabolism, which may be one of the potential mechanisms of ZSD for the treatment of acute pneumonia.

Based on the results of metabolomics analysis and data mining, it was hypothesized that the treatment of LPS‐induced acute pneumonia by ZSD was intricately linked to the sphingolipid metabolism, arachidonic acid metabolism, the JAK‐STAT signaling pathway, and the TLR signaling pathway. This observation underscores ZSD’s comprehensive regulatory role in ameliorating acute pneumonia, aligning with the characteristic multicomponent, multitarget, and multipathway mechanisms of TCM in disease treatment.

TCM treatment of disease does not primarily focus on “the similarities and differences of disease,” but instead on the differences of syndrome through the treatment of differential syndrome, thereby obtaining a better understanding of disease. Syndrome is an essential term in TCM theory, which results from careful analysis of all symptoms and signs. Syndrome differentiation‐based disease treatment can help us to identify the most suitable therapeutic approach [43, 44]. Future studies should develop animal models based on different TCM syndromes, combined with metabolomics strategies, to facilitate the interpretation and exploration of the core principles of personalized TCM and pave the way for a transformative revolution in healthcare. Furthermore, our multiomics analysis highlighted the critical role of specific targets, such as Sphk1; however, we did not investigate correlations between these targets and the levels of specific molecules within the pathways. Therefore, we will conduct molecular biology experiments to validate the S1PRs/SphK1 signaling pathway using a more inflammation‐sensitive animal model, such as C57BL/6 mice, thereby providing stronger mechanistic support for the therapeutic efficacy of ZSD on pneumonia.

## 5. Conclusion

This study systematically investigated the in vivo effects of ZSD on LPS‐induced acute pneumonia and elucidated its corresponding mechanism through an integrated approach of LC‐MS‐based metabolomics and GEO data mining. Biochemical analyses and H&E staining of lung tissues revealed that ZSD attenuated the levels of inflammatory mediators and mitigated pathological alterations in LPS‐challenged lung tissues. Lung tissue metabolome analysis showed that 37 and 81 significantly changed metabolites induced by LPS were selected as biomarkers in organic and aqueous lung tissue extracts, respectively. And 19 and 65 of them were reversed by ZSD treatment, respectively. Pathway analysis indicated that the therapeutic effect of ZSD may be related to the sphingolipid metabolism and AA metabolism. Furthermore, the integrated study of GEO data mining and metabolomics revealed an association between the TLR signaling pathway and the antiacute pneumonia effect of ZSD. Future investigations are required to validate the therapeutic mechanisms of ZSD in the context of acute pneumonia and to establish a robust foundation for its clinical application.

## Author Contributions

**Haipeng Tang and Zhiliang Sun**: methodology, software. **Haipeng Tang**: writing – original draft preparation. **Weibo Qin and Guangzhi Cai**: investigation, data curation. **Wenyi Gao and Jiyu Gong**: supervision. **Yang Wang**: supervision, writing – reviewing and editing.

## Funding

This study was funded by the China National Traditional Chinese Medicine Standardization Project (Grant ZYBZH‐Y‐JL‐25) and the Changchun University of Chinese Medicine (Grant 2023JQ05).

## Conflicts of Interest

The authors declare no conflicts of interest.

## Supporting Information

Additional supporting information can be found online in the Supporting Information section.

## Supporting information


**Supporting Information** Table S1: The information on purchased Chinese Materia Medica (CMM). Table S2: The parameters of different PLS‐DA models. Figure S1: The body weight (A) and lung index (B) in mice. Lung index of each group (lung index (%) = wet lung weight/body weight × 100). ##*p* < 0.01 versus control group,  ^∗^ *p* < 0.05,  ^∗∗^ *p* < 0.01 versus model group. Figure S2: The representative base peak chromatograms (BPC) of aqueous extract in three groups were acquired in positive ion mode (A, C, and E) and negative ion mode (B, D, and F). Figure S3: The representative base peak chromatograms (BPC) of organic extract in three groups were acquired in positive ion mode (A, C, and E) and negative ion mode (B, D, and F).

## Data Availability

The data that support the findings of this study are available from the corresponding author upon reasonable request.
