# Supplementary material for: Lung Tissue Metabolome Investigation Reveals the In Vivo Effects of Zhuye Shigao Decoction in an LPS‐Induced Acute Pneumonia Model in Mice
Source: Mediators Inflamm. 2026 May 11;2026:6034066. doi: 10.1155/mi/6034066 (PMC13158866; doi:10.1155/mi/6034066)
Supplement: Supplementary file 1 — Supporting Information Table S1: The information on purchased Chinese Materia Medica (CMM). Table S2: The parameters of different PLS‐DA models. Figure S1: The body weight (A) and lung index (B) in mice. Lung index of each group (lung index (%) = wet lung weight/body weight × 100). ##p < 0.01 versus control group, ∗ p < 0.05, ∗∗ p < 0.01 versus model group. Figure S2: The representative base peak chromatograms (BPC) of aqueous extract in three groups were acquired in positive ion mode (A, C, and E) and negative ion mode (B, D, and F). Figure S3: The representative base peak chromatograms (BPC) of organic extract in three groups were acquired in positive ion mode (A, C, and E) and negative ion mode (B, D, and F). [file MI-2026-6034066-s001.docx]

**Supplementary Material**

**Table S1** The information on purchased CMM

| No. | Latin name | English name | Chinese name | Batch No. |
| --- | --- | --- | --- | --- |
| 1 | *Phyllostachys nigra* (Lodd.) Munro var. *henonis* (Mitf.) Stapf ex Rendle. | Folium phyllostachydis henonis | Zhuye | BZ230701 |
| 2 | Gypsum Fibrosum | Gypsum fibrosum | Shigao | 1709271 |
| 3 | *Panax ginseng* C. A. Mey. | Ginseng radix et rhizoma | Renshen | 23072301 |
| 4 | *Ophiopogon japonicus* (L.f) Ker-Gawl. | Ophiopogonis radix | Maidong | 20190602 |
| 5 | *Pinellia ternata* (Thunb.) Breit. | Pinelliae rhizoma | Banxia | XH22082201 |
| 6 | *Glycyrrhiza uralensis* Fisch. | Glycyrrhizae radix et rhizoma | Gancao | HIQ23041501 |
| 7 | *Oryza sativa* L. subsp. *japonica* Kato. | Oryze semen | Jingmi | HL24012101 |

**Table S2** The parameters of different PLS-DA models

| **Groups** | **R2 Y（cum）** | **Q2（cum）** |
| --- | --- | --- |
| **Figure 3A** | 0.994 | 0.841 |
| **Figure 3B** | 0.893 | 0.737 |
| **Figure 3C** | 0.942 | 0.723 |
| **Figure 3D** | 0.921 | 0.741 |
| **Figure 4A** | 0.995 | 0.972 |
| **Figure 4B** | 0.823 | 0.617 |
| **Figure 4C** | 0.992 | 0.935 |
| **Figure 4D** | 0.975 | 0.840 |


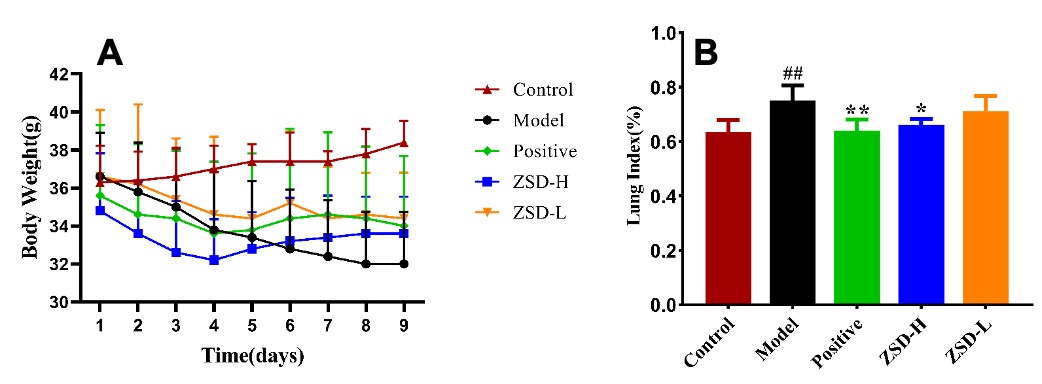


**Figure S1** The body weight (A) and lung index (B) in mice. Lung index of each group (lung index % = wet lung weight/body weight × 100). ##P<0.01 vs control group, *P<0.05, **P<0.01 vs model group.


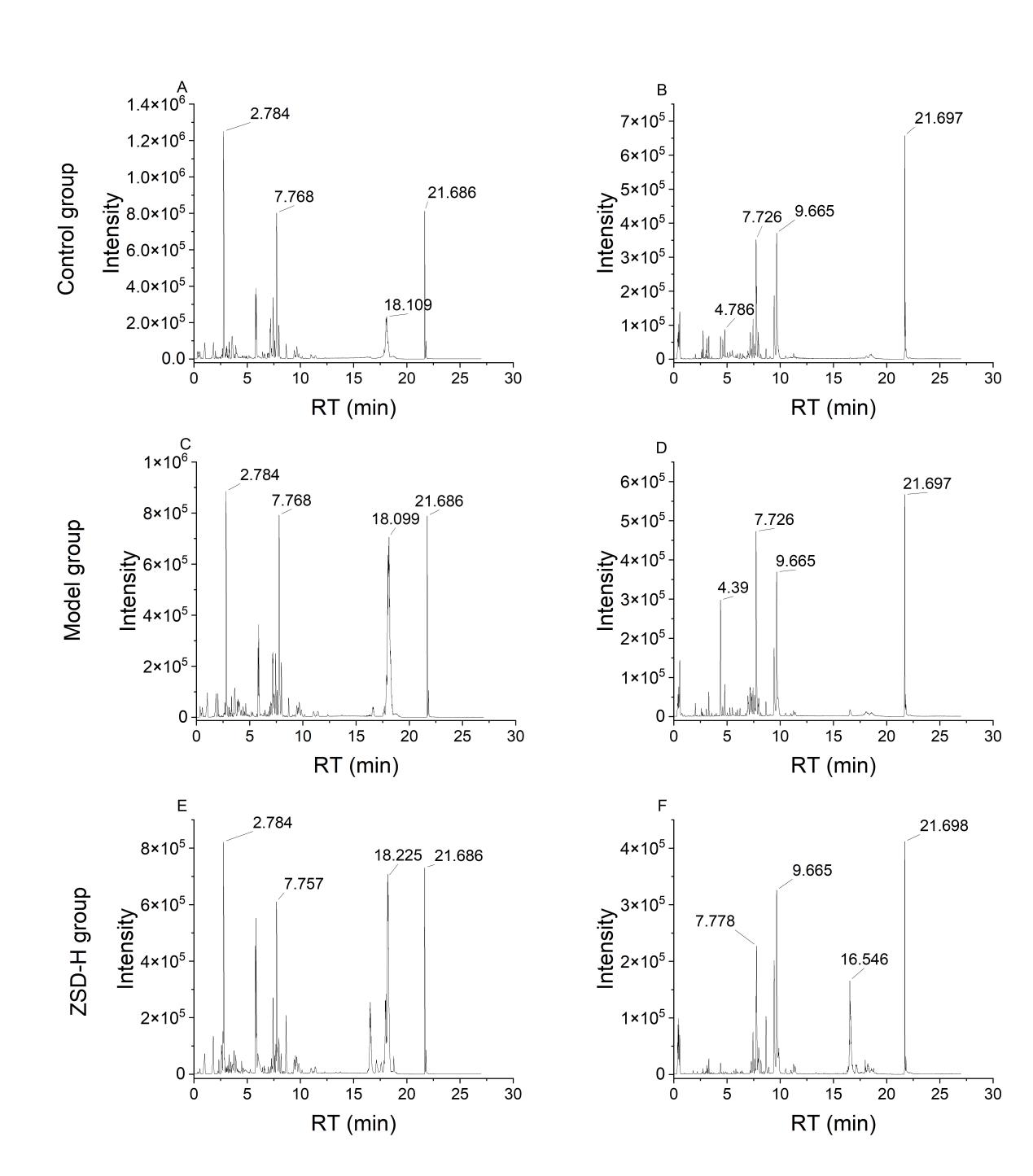


**Figure S2** The representative base peak chromatograms (BPC) of aqueous extract in three groups were acquired in positive ion mode (A, C, and E) and negative ion mode (B, D, and F)


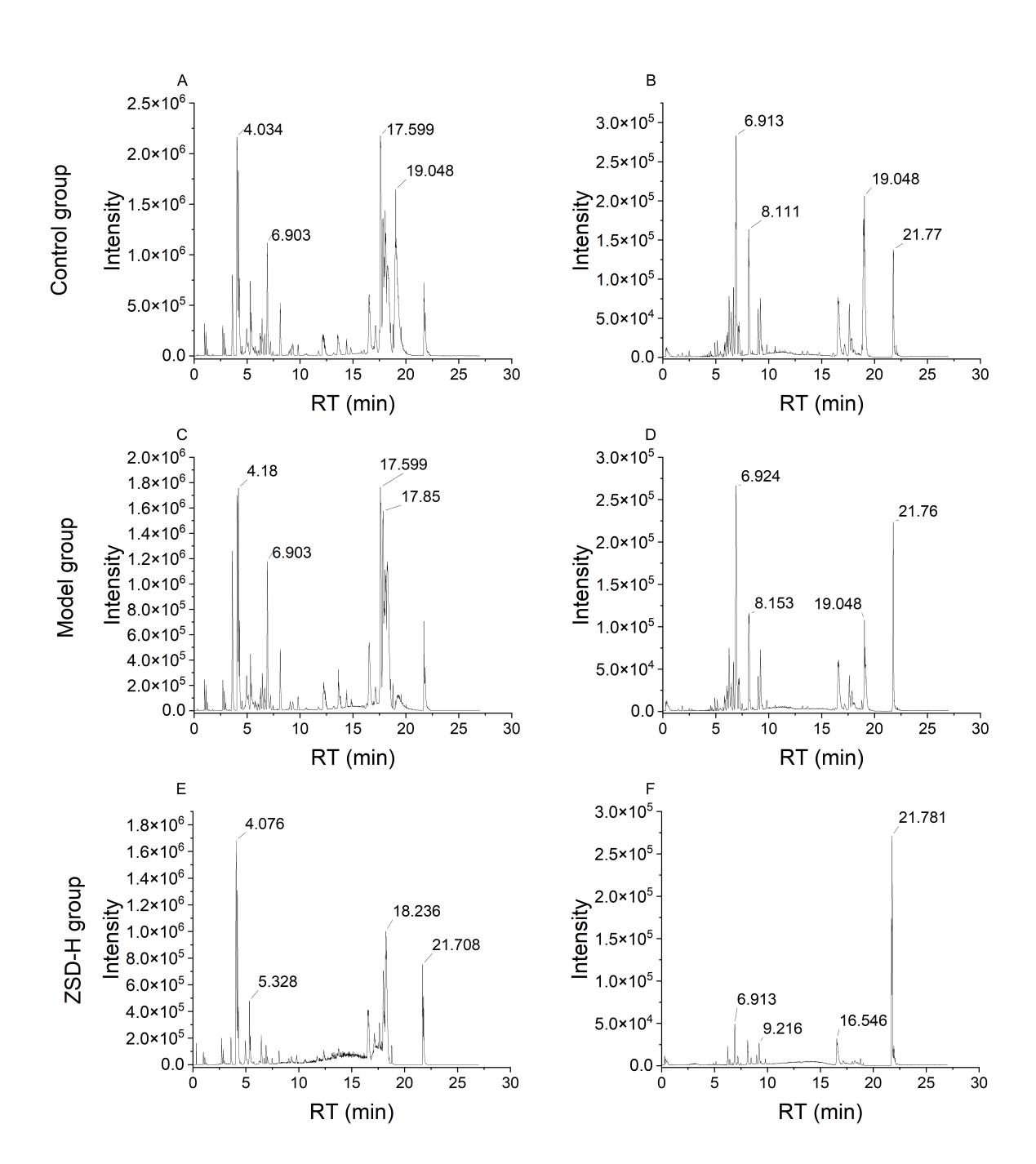


**Figure S3** The representative base peak chromatograms (BPC) of organic extract in three groups were acquired in positive ion mode (A, C, and E) and negative ion mode (B, D, and F)
